# *Helicobacter Pylori*: Prevalence and Relationship with Abdominal Pain in School Children in Makkah City, Western Saudi Arabia

**DOI:** 10.4103/1319-3767.45359

**Published:** 2009-04

**Authors:** Abdulwahab M. A. Telmesani

**Affiliations:** Department of Pediatrics, Medical College, Umm Al-Qura University, Makkah, Saudi Arabia

**Keywords:** Abdominal pain, children, *H. pylori*, Saudi Arabia, urea breath test

## Abstract

**Background/Aim:**

The published data on *Helicobacter pylori* (*H. pylori*) prevalence and its relationship with abdominal pain in Saudi Arabia is scarce. This study was carried out to determine the prevalence of *H. pylori* and its relationship with chronic recurrent abdominal pain (RAP) among school students in Makkah City, Saudi Arabia.

**Materials and Methods:**

Three hundred and fourteen school students, 103 at the intermediate level (grades 7-9) aged 12-15 years and 211 at the secondary level (grades 10-12) aged 15-18 years were tested for *H. pylori.* Urea breath test (UBT) was used for this purpose. Children with chronic RAP were identified as per the Apley criteria.

**Results:**

Overall, the UBT was positive in 86/314 (27.4%) students. It was positive in 45/103 (43.7%) intermediate school students and 41/211 (19.4%) secondary students. Out of the 55 students with chronic RAP, 40 (73%) were positive for *H. pylori*. Further, 62.9% and 82.1% were positive among the intermediate and secondary school students with RAP, respectively. The overall and specific odds ratios of RAP were 12.35 [95% confidence interval (C.I.) 6.30-24.22] and 10.40 (95% C.I. 1.75-11.73) for the intermediate school students and 22.69 (95% C.I. 7.99-64.44) for the secondary school students.

**Conclusion:**

The prevalence of *H. pylori* among the school children in Makkah, Saudi Arabia, is relatively low compared with developing countries. The prevalence was found to be higher among the younger age group. Further, there was a significant relation between *H. pylori* infection and RAP among the school students.

The causal association of ***Helicobacter pylori*** to peptic disease and gastric cancer is well established, yet there is insufficient evidence to conclude the benefit of population screening for ***H. pylori***.[1] Studies point toward a relation between the low socioeconomic status and the high rate of ***H. pylori*** infection. Epidemiology of ***H. pylori*** infection demonstrated a high prevalence in developing than in developed countries, where it reached up to 90%.[2] This prevalence was found to be high among the immigrants compared with the local population in the developed countries.[3–4] A low prevalence rate has been reported in the developed countries. Herbarth ***et al.*** reported a prevalence of 6.5% among school children in Germany.[5] Improving the living conditions lowers the rate of infection. The decrease in the rate of infection of ***H. pylori*** in southern China from 1993 to 2003 was attributed to an improvement in the socioeconomic conditions.[6] In the Middle East countries like Turkey, the prevalence was 44% in children and in other studies, the prevalence was up to 89%.[7–8] In Saudi Arabia, ***H. pylori*** was found in 67-87% of the children with peptic disease.[9–10] A study of ***H. pylori*** among children with chronic diseases in Jeddah, Saudi Arabia, showed a prevalence of 23.6%.[11] The relationship of ***H. pylori*** with recurrent abdominal pain (RAP) is still controversial. ***H. pylori*** has been found in 60.3% of the children with RAP who benefited from eradication therapy.[12] On the other hand, other studies failed to show a relation between ***H. pylori*** infection and RAP.

This study was undertaken to verify the prevalence of ***H. pylori*** and its relationship with RAP among school students in Makkah City, Saudi Arabia.

## MATERIALS AND METHODS

This cross-sectional study was conducted at a boys school in Makkah City, Makkah region, Western Saudi Arabia. The school has two levels: intermediate (grades 7-9), which includes children aged 12-15 years, and secondary (grades 10-12), which includes children aged 15-18 years. Informed consent was obtained from parents. A self-administered questionnaire was used to acquire information on allergy toward the test material and the recent use of antibiotics (exclusion criteria). Children with chronic abdominal pain were identified according to the Apley criteria (at least three episodes of abdominal pain severe enough to affect activity over a period of at least 3 months). Three hundred and sixteen students were qualified to take the urea breath test (UBT): 103 intermediate and 213 secondary students. The UBT used was Heliprobe 14C UBT (Noster Heliprobe System AB, Stockholm, Sweden). The test sensitivity exceeds 97%, with a specificity of 95%.[13–14] The samples were taken from the students on site at the school. Heliprobe 1 micro Curie 14C was given as a capsule or dissolved in juice or water. After 10 min, the students were asked to blow (only exhaled)/breathe into the Heliprobe breathCard, provided by the Heliprobe system, until the card indicator of the breath card changed from orange to a yellow color. The breathCards were collected and taken to the lab for analysis. The breathCards were analyzed by the Heliprobe analyzer provided by the Heliprobe system. The results were calculated using grades “0, 1, or 2” (Grade 0: not infected; Grade 1: borderline; and Grade 2: infected). The borderline required a repeat test.

## STATISTICAL ANALYSIS

Statistical package for social sciences (version 13.0) was used to process the data and analyze it. Analyses of the data included calculating the odds ratios (O.R.) and 95% confidence intervals (95% C.I.) and running χ^2^ tests. All these were applied to assess the statistical relationship. A ***P***-value < 0.05 was considered statistically significant.

## RESULTS

The UBT test was positive in 86/316 (27.2%) and borderline in only two secondary school candidates. The two borderline subjects failed to respond to retesting and were excluded, which made the study sample to be based on 314 candidates. Of the 86 UBT positives, 45 (43.7%) were intermediate school students and 41 (19.4%) were secondary school students. RAP was found in 55/314 (17.5%): 27 (26.2%) intermediate and 28 (13.3%) secondary school students. Further, of these 55 students who had RAP, 40 (73%) were UBT positive: 17/27 (63.0%) and 23/28 (82.1%) intermediate and secondary school students, respectively. Therefore, in general, the children infected with ***H. pylori*** are 12.3 times at risk of RAP compared with those not infected (O.R. = 12.35, 95% C.I. (6.30-24.22), ***P*** < 0.000) [Table 1]. Furthermore, for the intermediate school students, the infected ones with ***H. pylori*** are 10.4-fold at risk of RAP than those not infected (O.R. = 10.40, 95% C.I. (1.75-11.73), ***P*** = 0.001) [Table 2], while the infected students at the secondary school are almost 23 times at risk of RAP than those not infected (O.R. = 22.69, 95% C.I. (7.991- 64.443), ***P*** < 0.000) [Table 3].

**Table 1:** The relationship between RAP and UBT results in general

	Children with RAP	Children without RAP	Total
Positive UBT	40 (72.7)	46	86 (27.4)
Negative UBT	15	213	228
Total	55 (17.5)	259	314

RAP = Recurrent abdominal pain, UBT = Urea breath test, Figures in parentheses are in percentages

**Table 2:** The relationship between RAP and UBT results among the intermediate school children

	Children with RAP	Children without RAP	Total
Positive UBT	17 (63.0)	18	35 (37.6)
Negative UBT	10	58	68
Total	27 (29.0)	76	103

RAP = Recurrent abdominal pain, UBT = Urea breath test, Figures in parentheses are in percentages

**Table 3:** The relationship between RAP and UBT results among the secondary school children

	Children with RAP	Children without RAP	Total
Positive UBT	23 (82.1)	30	53 (25.7)
Negative UBT	5	153	158
Total	28 (13.6)	183	211

RAP = Recurrent abdominal pain, UBT = Urea breath test, Figures in parentheses are in percentages

## DISCUSSION

This cross-sectional study included intermediate and secondary school students in Makkah City, Western Saudi Arabia, using 14C UBT. It is considered to be the first, local, study on healthy school students using UBT. The prevalence of 27.4% is relatively low compared with developing countries. The study concurs with the study in the neighbouring Jeddah City, utilizing serology testing, which was 23.6%.[11] The low prevalence might be related to a higher socioeconomic status relative to the developing countries. A study from Sri Lanka reported a similar low prevalence of 27.7% with unexplained reasons.[15] This study also showed a higher prevalence among the intermediate school students of 43.7% compared with 19.4% among the secondary school children. This is contrary to studies that show an increase in the prevalence with age.[16,17]

The UBT was chosen because it is not invasive and has a high sensitivity and specificity.[13–14] The saliva-based ***H. pylori*** test, although not invasive, has a lesser sensitivity (74-80.9%) and specificity (67-95.7%) than the UBT.[18,19] Stool antigen, a non-invasive test with 91.5% sensitivity and 89.6% specificity.[20] However, the problem was verification of the sampling. Serology, on the other hand, is an invasive study that may affect the parental consent and ethical clearance. In addition, serology leads to at least four times as many false results as the UBT and stool antigen test.[21]

The role of ***H. pylori*** and adult peptic ulcer in Saudi Arabia and internationally has been established.[22–24] Similarly, a high prevalence among children with peptic diseases has been reported.[9–10,25–26] The present study shows UBT to be positive in 63.6% of children identified to have RAP while the overall prevalence was 27.3%. Further, UBT was positive in 63.0% and 82.1% of the intermediate and secondary students, respectively. However, RAP was more prevalent among the intermediate than among the secondary school students (26.2% vs 13.3%). Iron deficiency anemia and short stature have been linked to ***H. pylori*** infection whereas the link with RAP is still not settled.[27–29] A prospective study revealed an association of ***H. pylori*** and RAP in younger children.[30] Children with RAP benefited from ***H. pylori*** eradication.[12] Nakayama ***et al.*** recommended that children with RAP who meet the Rome II criteria should be tested for ***H. pylori*** whereas Vibeke from Denmark showed no causal relationship between RAP and ***H. pylori***.[31–32] The Canadian ***H. pylori*** study group and Sherman concluded that RAP is not an indication for testing for ***H. pylori***.[33–34] The present study reveals a high prevalence of ***H. pylori*** in children with RAP. The study revealed that infected children are 12 times at risk of RAP and the risk is higher, 23 times, in older children. There is not enough data to warrant population ***H. pylori*** screening. However, with the documented public health implications, ***H. pylori*** requires a wide range of analytical epidemiological studies in the form of prospective and/or randomized controlled trials.

It is recognized that this study has some limitations, of which this is a cross-sectional study and not a random sample, is carried out in males, and the socioeconomic status of the studied students was not include. To overcome these limitations, a larger study that excludes all the limitations should follow.

In conclusion, the prevalence of ***H. pylori*** among students of an intermediate-secondary school in Saudi Arabia is lower than those reported from the neighbouring countries and developing countries. There was a high prevalence of ***H. pylori*** among the school students with RAP. The relationship between ***H. pylori*** infection and RAP was statistically significant. Further studies including female school students and nation-wide multidimensional ones are required. The causal association and the response to eradication of ***H. pylori*** and RAP require investigation and the benefit of public screening needs further study.
